# Exposure to Fumes of a Vegetable Margarine for Frying: Respiratory Effects in an Experimental Model

**DOI:** 10.1021/acsomega.3c03340

**Published:** 2023-08-22

**Authors:** Arif H. Cimrin, Aylin Ozgen Alpaydin, Seda Ozbal, Melis Toprak, Osman Yilmaz, Funda Uluorman, Bekir Ugur Ergur, Duygu Gurel, Sait C. Sofuoglu

**Affiliations:** † Department of Pulmonary Medicine, Faculty of Medicine, Dokuz Eylul University, 35340 Izmir, Türkiye; ‡ Department of Histology and Embryology, Faculty of Medicine, Dokuz Eylul University, 35340 Izmir, Türkiye; § Department of Environmental Engineering, Izmir Institute of Technology, Urla, 35430 Izmir, Türkiye; ∥ Multidisciplinary Animal Laboratory, Faculty of Medicine, Dokuz Eylul University, 35340 Izmir, Türkiye; ⊥ Department of Histology and Embryology, Faculty of Medicine, Kyrenia University, 99320 Kyrenia, Cyprus; # Department of Medical Pathology, Faculty of Medicine, Dokuz Eylul University, 35340 Izmir, Türkiye

## Abstract

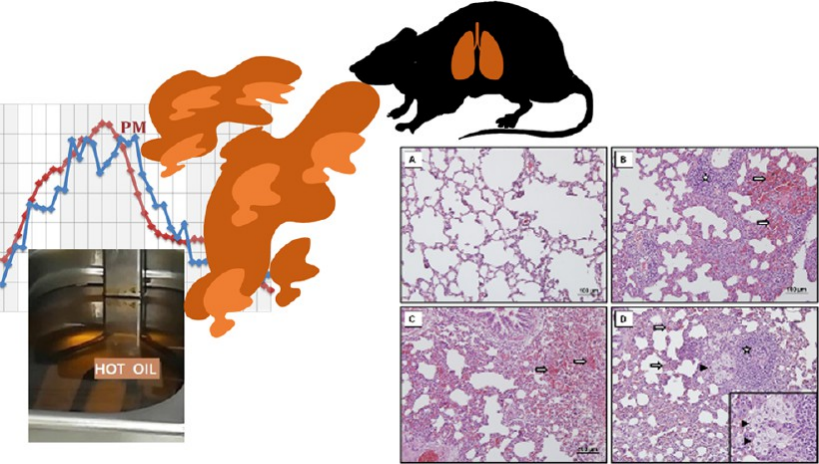

Deep frying is one of the strongest emission sources into indoor air. A vegetable margarine has recently been used in commercial kitchens. This study investigated the respiratory effects of exposure to its fumes in an experimental model. A setup with glass chambers was constructed. A chamber housed a fryer. The fumes were transported to the other chamber where 24 Wistar albino rats were placed in four randomized groups: acute, subacute, chronic, and control for the exposure durations. PM_10_ concentration in the exposure chamber was monitored to ensure occupational levels were obtained. Sacrification was performed 24 h after exposure. Lung, trachea, and nasal concha specimens were evaluated by two blinded histologists under a light microscope with hematoxylin–eosin. Mild mononuclear cell infiltration, alveolar capillary membrane thickening, alveolar edema, and diffuse alveolar damage, along with diffuse hemorrhage, edema, and vascular congestion in the interstitium were observed in the acute and subacute groups, and were overexpressed in the chronic group, whereas normal lung histology was observed in the control group. The results indicate that exposure to fumes of vegetable margarine for frying in commercial kitchens may cause pulmonary inflammation that becomes severe as the duration of the exposure increases.

## 1 Introduction

Cooking is one of the important sources of indoor air pollution.^1^ Frying is a common cooking method, especially in industrial kitchens, and brought forward due to its strong emission potential.^2−6^ Corn, safflower, vegetable, and olive oils are used for frying.^7^ Fast food restaurants are increasingly preferred in nutrition because they are time-saving and inexpensive. Most of the products are fried in these restaurants. An increasing number of restaurants and increasing demand for their products mean more people are exposed to frying fumes and other emissions for longer periods. Frying at high temperatures results in carcinogenic fumes^8^ that contain particulate matter (PM), aldehydes, volatile organic compounds (VOCs), and polycyclic aromatic hydrocarbons (PAHs).^8−10^ Epidemiological studies indicate that cooks and bakers have higher carcinogenic risks.^11^ Nonsmoking women have been shown to have a higher carcinogenic risk in East Asia.^12,13^ PAHs and aldehydes have mutagenic effects.^14,15^ Aldehydes diffuse into cells, causing damage by reacting with macromolecules such as DNA.^16^ Similar to PAHs and aldehydes, some of the VOCs are carcinogenic substances that cause mucous membrane irritation.^17^ Some of the aldehydes, such as acrolein and formaldehyde, are strong irritants.^18^ Dienaldehyde was reported to cause increased reactive oxygen (ROX) products, proinflammatory cytokine tumor necrosis factor, and interleukin-1β (IL-1β) on the human bronchial cell line.^19^ Decreased cell viability, oxidative stress, inflammation, and apoptosis were also reported in Beas-2B cells by heated peanut oil fumes,^20^ while healthy cell damage was reported to occur even at a low-dose exposure to cooking oil fume contaminants, i.e., heterocyclic aromatic amines and aldehydes.^21^

Inhalation of PM in cooking fumes was reported to cause pulmonary, cardiac, reproductive, renal, and dermal toxicity.^16^ Type of frying oil, temperature, time, type of food, and amount are determinants of the size and concentration of particles in the fumes.^22^ Ultrafine particles (UFP, PM_0.1_) dominate in terms of number concentration, whereas in terms of mass concentration, the majority of PM_10_ consist of fine particles (PM_2.5_), while sub-micron particles (PM_1.0_) dominate PM_2.5_.^23,24^ The surface area available for sorption of organic compounds, such as PAHs, increases with decreasing size of the particles, which have higher ROX and oxidative stress formation potential.^25−27^ UFP have higher peripheral lung accumulation rates than those of larger particles,^28^ which reduces the capacity of alveolar macrophages to remove exogenic particles.^27^ The increase in alveolar macrophages was reported to be an indicator of occupational pulmonary irritation in fast food and grill kitchen workers.^29^ PM was associated with premature death, asthma exacerbation, chronic bronchitis, and effects on the immune system.^26,30^ Cooking fumes were related to increased frequency of respiratory symptoms in kitchen workers^31,32^ and decreased lung function.^33^ Short-term functional changes were observed after exposure to cooking fumes in an experimental study.^32,34^ Exposure to Chinese-style open-wok cooking fumes, which are rich in PM, was found to have a strong relation to rhinitis.^35^

The time spent frying was reported to be a determinant for increased personal total dust exposure in large-scale and European kitchens, where group geometric mean and individual personal sample concentrations reached 320 and 3900 μg/m^3^, respectively.^36^ It has been reported that PM_2.5_ concentrations may exceed maximum contaminant levels due to emissions of commercial kitchens^7^ where indoor air pollutant concentrations exceed those of residential kitchens.^1^ The difference may be attributable to the differences in foods and styles of cooking.^7^ Deep-fried foods are popularly consumed in commercial establishments where a type of vegetable margarine for frying is used in Turkey. We have studied the indoor air quality in the kitchen of such a small establishment before, during, and after frying events.^37^ Results of our study showed that considerably high levels of occupational exposure to PM_10_ occur in the kitchen during frying, while VOC and aldehyde concentrations were also increased during frying, but not as sharply as for PM. CO_2_ concentrations, on the other hand, were not increased.

There is strong evidence that the respiratory toxic effects of exposure to cooking oil fumes include both airway and parenchymal damage, and that this is associated with oxidative stress, which was based on findings observed after 30 days of smoke exposure.^38^ It was shown that apoptotic cytokines increased significantly along with the increase in proinflammatory cytokines. In addition to increased inflammatory cell infiltration in the tissue, goblet cell hyperplasia and increased fibrosis were detected. We conducted an experimental study based on two cases of fast food cooks diagnosed with alveolar damage and asthma, associated with occupational exposure to frying oil fumes in our clinic, and based on the literature on the toxic effects of frying fumes. With this model, we aimed to investigate the nasal, tracheal, and respiratory parenchymal effects of acute, subacute, and chronic exposure models in mice exposed to the fumes of the frying margarine used in industrial kitchens in Turkey.

## 2 Materials and Methods

### 2.1 Occupational Concentrations

Our previous study reported methods employed for the determination of indoor air quality in a small establishment that uses deep-frying margarine made of palm oil with dimethylpolysiloxane as an antioxidant and antifoaming additive.^37^ The establishment serves mainly lunch. Several foods (potatoes, chicken, beef) were fried in a 3 L container at 160–180 °C in a naturally ventilated kitchen whose doors and windows were kept closed during frying.

The measurements were conducted in two 1-week campaigns. Each campaign consisted of three days that started 1.5 h before and ended 1.5 h after lunch. Measurements were made in three time periods: before, during, and after frying to determine the increase and decrease in pollutant concentrations with reference to the background. The first campaign was for the regular operation, while the second campaign was conducted during an out-of-service period to investigate the effect of the amount of fried potatoes on the kitchen indoor air concentrations: 1.25 kg denoting the regular operation, 2.5 and 3.75 kg on the first, second, and third days, respectively.

Samples of VOCs and aldehydes were collected in before-, during-, and after-frying periods, while samples of PM_2.5_ were collected in the whole 4 h due to concerns that shorter sampling would not be sufficient for gravimetrical measurement. In the meantime, continuous monitoring was conducted for total VOCs (TVOC), PM_10_, CO, and CO_2_. Stationary sampling/monitoring was conducted 50 cm away from the fryer at 1.5 m height. Further details of the methods employed can be found in our previous study.^37^

### 2.2 Exposure Chamber

**Figure 1:**
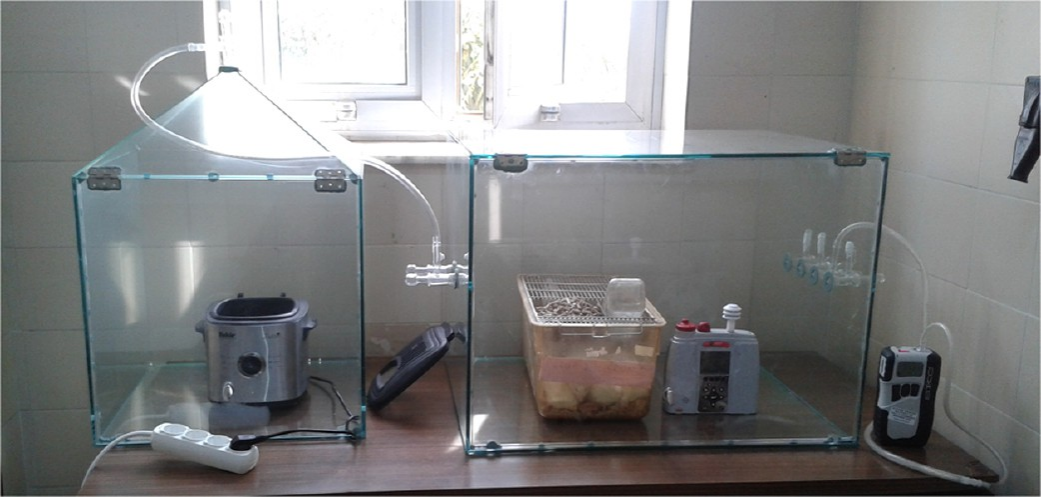
Glass exposure chamber custom-built for the experiment.

An exposure system consisting of two parts was made of glass (Figure 1). The first unit, 50 cm × 50 cm × 50 cm (W × L × H) with a 15 cm roof above, was built to house a fryer with a 1 L oil container. An exhaust on the tip of the roof was connected to the second unit with a 0.25 in. inner-diameter Tygon tubing. The second unit, 75 cm × 50 cm × 50 cm (W × L × H), was built to house the rats and the monitoring device. This unit was equipped with a front door and eight valves. The tubing from the first unit was connected to one of the valves on the left. One of the valves on the right was connected to a vacuum pump to sustain 1 air change per hour in the chamber.

### 2.3 Monitoring and Standardization of Exposure

In our previous study to determine occupational exposure levels,^37^ the average peak PM_10_ concentration of the during-frying period was determined to be 1583 μg/m^3^ for regular operation in the studied establishment, whereas the average during-frying concentration was 4037 μg/m^3^ in the second campaign with increased amount of fried potatoes. Therefore, roughly, the overall average value of the whole study, i.e., 2500 μg/m^3^, was selected as the exposure concentration in the experiment so that a realistic occupationally relevant concentration is investigated. Preliminary runs were made to determine how long the margarine needed to be heated to achieve and sustain the decided experimental concentration. As a result, the fryer was heated to 180 °C 1 h before each exposure session, providing PM_10_ concentrations of 2500 ± 250 μg/m^3^ during the sessions. Margarine was put into the fryer to fill the container up to the maximum line when melted, which melts at room temperature and is kept in solid form in the refrigerator at 4 °C, and topped when it dropped to the minimum line in the container.

### 2.4 Animal Model

The study protocol was approved by the Ethical Committee of Dokuz Eylül University Medical School (permit no: 31-2010). Male adult Wistar rats (*n*:24) (Dokuz University School of Medicine, Izmir, Türkiye) weighing 200–250 g were used. Animals were housed in an appropriate cage on a 12 h light/12 h dark cycle with free access to standard laboratory food and tap water. The animals were allowed to habituate to the housing facilities for at least 1 week before the starting of experiments. They were divided into four groups of six animals each.

The four groups of six rats were formed by random selection: group 1: acute exposure (120 min), group 2: subacute exposure (360 min), group 3: chronic exposure (120 min daily for three weeks), group 4: controls. Whole-body exposure was applied. Rats in groups 1, 2, and 3 were sacrificed right after their respective exposure periods, while the controls were sacrificed along with group 3. All groups were kept in separate cages and brought into the exposure chamber at the start of the session.

### 2.5 Histological Assessment

The head, trachea, and lung tissues were removed after sacrification and fixed in 10% buffered neutral formalin for three days. Routine tissue follow-up was initiated after the fixation of the trachea and lung tissues. After washing under a stream of water for a night to remove the fixative, the tissues were kept in the oven for 20 min at 60 °C and then passed through a series of increasing ethyl alcohol: 70, 80, and 96%. Dehydration was followed in four changes of 20 min in acetone, then two changes of 30 min in xylol for transparency, and two changes of paraffin immersion for 1 h, all in a 60 °C oven, before embedding in paraffin blocks. A rotary microtome (RM 2255, Leica, Germany) was used for taking 5 μm sections and stained with hematoxylin–eosin (H–E).

The fixed subject heads were decalcified in EDTA for 2 months. Tissue blocks were removed with two perpendicular sections extending from the anterior nasal cavity to the hard palate. They were embedded in paraffin blocks for routine tissue follow-up after washing under a stream of water for a night. A rotary microtome (RM 2255, Leica, Germany) was used for taking 5 μm sections. Nasal conchae sections of each subject were stained with H–E to evaluate the general histomorphological features of the tissue.

#### 2.5.1 Hematoxylin–Eosin Staining

The sections were left in the oven at 60 °C for 2 h for deparaffinization. Then, they were subjected to xylene, first in the oven for 20 min and then two times for 10 min. Rehydration was followed with two changes of absolute and in a series of decreasing percentages of 96–70% alcohol. The sections were stained with hematoxylin (01562E, Surgipath, Bretton, Peterborough, Cambridgeshire) for 10 min after rinsing with distilled water. After staining, they were washed in the stream for 10 min to remove excess paint from the tissue and then stained with eosin (01602E, Surgipath, Bretton, Peterborough, Cambridgeshire) for 2 min. The sections were passed through 70, 80, and 96% alcohol in a series of two, and absolute alcohol, followed by three changes of xylene for 20 min for transparency before closing with Entellan (UN 1866, Merck, Darmstadt, Germany).

##### 2.5.1.1 Lung Tissue

At least 20 lung areas in three nonoverlapping lung sections per subject were investigated by skipping the areas with large vessels and airways to evaluate parenchymal changes by light microscopy. General morphological changes (alveolar structures, inflammation, alveolar septum, alveolar macrophage and neutrophil, and hemorrhage, edema, and congestion in the parenchyma) were evaluated with the H–E stained sections, while changes in the alveolar septum and connective tissue changes in parenchyma were evaluated with the Masson’s trichrome stained sections. Each lung was evaluated by looking at alveolar structures, inflammation, increased capillary permeability, thickening of alveolar septa, increase in alveolar macrophage and neutrophil counts, and hemorrhage, edema, and congestion in the parenchyma. These findings were scored with 0, 1, 2, 3, and 4 for no, light, mild, obvious, and very obvious observations, respectively. Then, averages were calculated for comparison.^39^

##### 2.5.1.2 Trachea Tissue

The sections stained with H–E were evaluated semiquantitatively for epithelium (erosion and inflammation), basement membrane (normal or thickened), and lamina propria (congestion, hemorrhage, and inflammation) to assess tracheal damage. Histological parameters were scored with 0 (no change), 1 (light), 2 (mild), and 3 (obvious). Then, averages were calculated for comparison.^40^

#### 2.5.2 Image Analysis Methods

The H–E staining sections were evaluated by two investigators blinded to the study by light microscopy (Olympus BX-50 Tokyo, Japan). High-resolution digital images were produced with a computer equipped with an Olympus DP-71 (Japan) camera. The images were assessed using the digital image analysis software (UTSCSA Image tool version 3.0 for Windows, Texas).

### 2.6 Statistical Analysis

All data were presented as mean ± SEM. Statistical testing for differences between two and multiple groups was conducted with the Mann–Whitney *U*-test and Kruskal–Wallis test, respectively, using SPSS 25.0. A *p*-value of < 0.05 was considered statistically significant.

## 3 Results

### 3.1 Histomorphology of Lung Parenchyma

**Figure 2:**
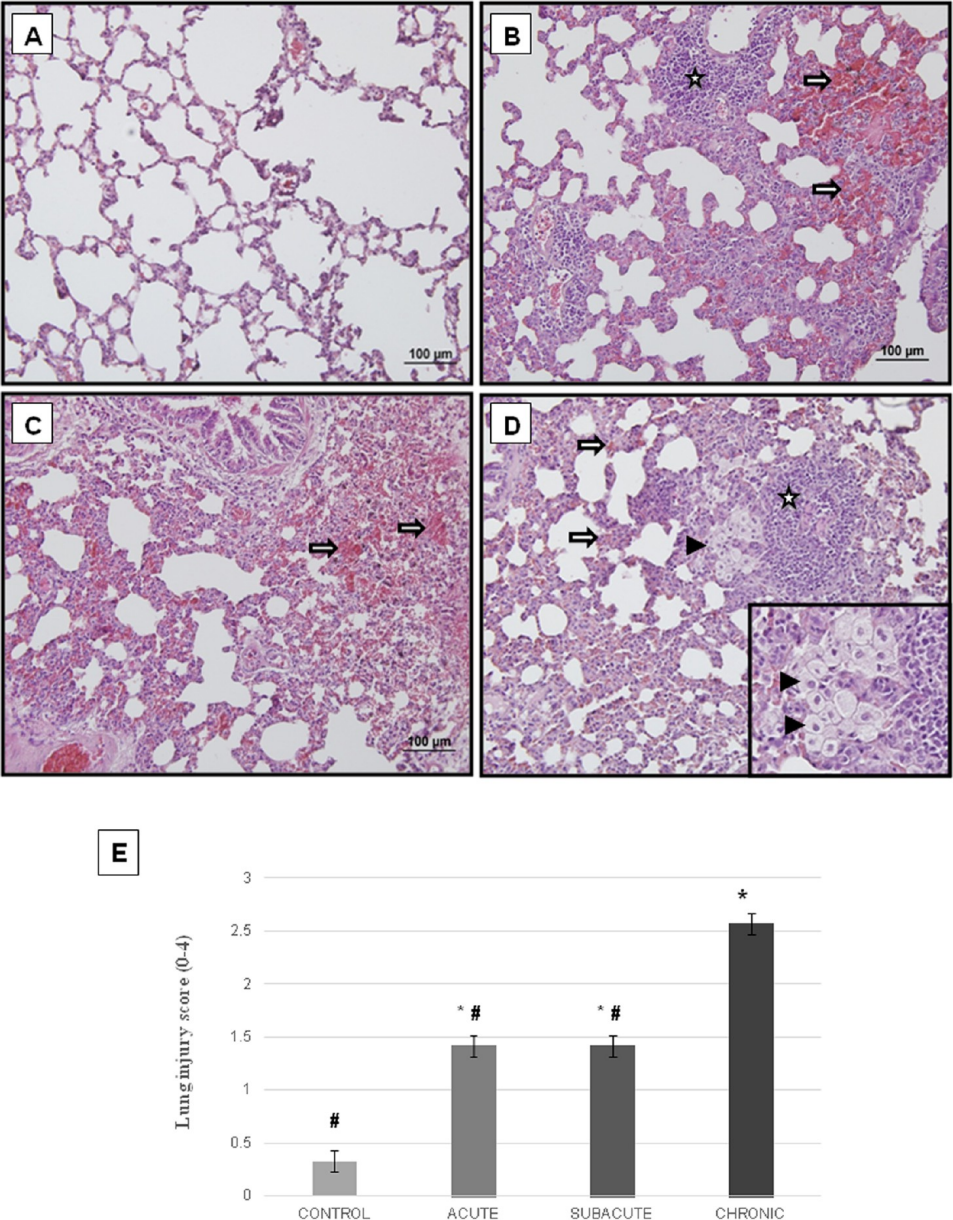
Histomorphological damage in the lung tissue of the experimental groups. Representative light microscopic images of H–E staining in the control group (A), acute group (B), subacute group (C), and chronic group (D). The hollow star indicates chronic pulmonary inflammation that expands the interstitium, (⇒) indicates erythrocyte extravasation, and (▶) indicates the foci of collection of septal and intra-alveolar foamy histiocytes. The scores of lung histomorphological damage (E). *p < 0.05 vs control, ^**#**^*p* < 0.05 vs. chronic group. Error bars show standard error of the mean.

Figure 2 demonstrates the histological findings of each group in the lung tissue of animals exposed to heated frying-margarine fumes for the control, acute, subacute, and chronic exposure groups.

#### 3.1.1 Control Group (*n* = 3)

The structure of the lung tissue of the control group was evaluated as normal. Alveolar structures were normal. There was no increase in inflammation, capillary permeability, thickening in alveolar septa, and number of alveolar macrophages. No findings related to hemorrhage, edema, and congestion were found in the parenchyma (Figure 2A).

#### 3.1.2 Acute (*n* = 7) and Subacute (*n* = 7) Exposure Groups

Extensive lung damage was observed compared to the control group. A small amount of mononuclear cell infiltration and an increase in capillary permeability were found. Histomorphological evaluation of alveoli revealed thickening of alveolar septa, alveolar edema, and diffuse alveolar damage. Diffuse hemorrhage, mononuclear cell infiltration, edema, and vascular congestion were detected in the parenchyma (Figure 2B,C, respectively).

#### 3.1.3 Chronic Exposure Group (*n* = 7)

Higher lung damage was observed in the chronic exposure group compared to the acute and subacute groups. Widespread mononuclear cell infiltration and increased capillary permeability were found. Obvious thickening of alveolar septa, alveolar edema, and diffuse alveolar damage were observed. Diffuse hemorrhage, mononuclear cell infiltration, edema, and vascular congestion were increased in the parenchyma (Figure 2D).

The scores of histomorphological damage in the lung tissue increased significantly in the acute, subacute, and chronic groups when compared to the control group (*p* = 0035, *p* = 0035, and *p* = 0012, respectively). The scores of the chronic group were significantly higher when compared to the acute and subacute groups (*p* = 0006 and *p* = 0006, respectively) (Figure 2E).

### 3.2 Histomorphology of Trachea

**Figure 3:**
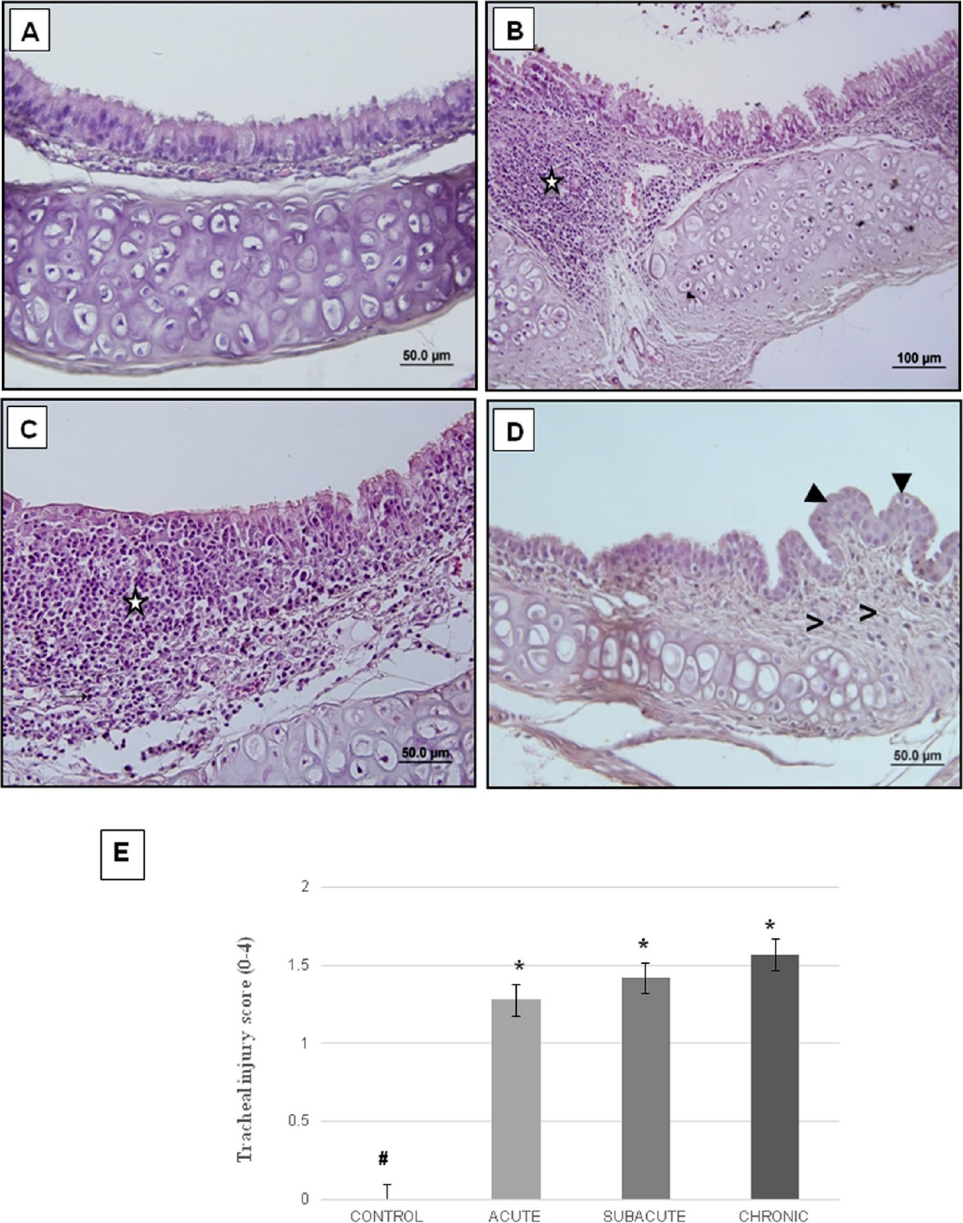
Histomorphological damage in the trachea tissue of the experimental groups. Representative light microscopic images of H–E staining in the control group (A), acute group (B), subacute group (C), and chronic group (D). (★) indicates chronic inflammation that also infiltrates the respiratory epithelium, (▶) indicates mucosal papillation and squamous metaplasia, and (**>**) indicates fibrosis. The scores of tracheal histomorphological damage (E). ^**#**^*p* < 0.05 vs control group. Error bars show standard error of the mean.

Figure 3 demonstrates the histological findings of each group in the trachea tissue of animals exposed to heated frying-margarine fumes for the control, acute, subacute, and chronic exposure groups.

#### 3.2.1 Control Group (*n* = 3)

The structure of the trachea tissue of the control group was evaluated as normal. Respiratory epithelium, lamina propria, and cartilage structures were normal, and no epithelial changes and inflammation were observed (Figure 3A).

#### 3.2.2 Acute (*n* = 7) and Subacute (*n* = 7) Exposure Groups

Extensive tracheal injury was observed compared to the control group. Widespread chronic inflammation in the lamina propria and an increase in diffuse mononuclear cell infiltration were observed (Figure 3B,C, respectively).

#### 3.2.3 Chronic Exposure Group (*n* = 7)

Tracheal damage was more common compared to the acute and subacute exposure groups. Papillation, squamous metaplasia, and mononuclear cell infiltration were observed. Chronic inflammation and fibrosis were also detected in the lamina propria (Figure 3D).

The scores of histomorphological damage in the trachea tissue increased significantly in the acute, subacute, and chronic groups when compared to the control group (*p* = 0009, *p* = 0011, and *p* = 0011, resp.). No significant difference was observed between the acute, subacute, and chronic groups (Figure 3E).

### 3.3 Histomorphology of Nasal Conchae

**Figure 4:**
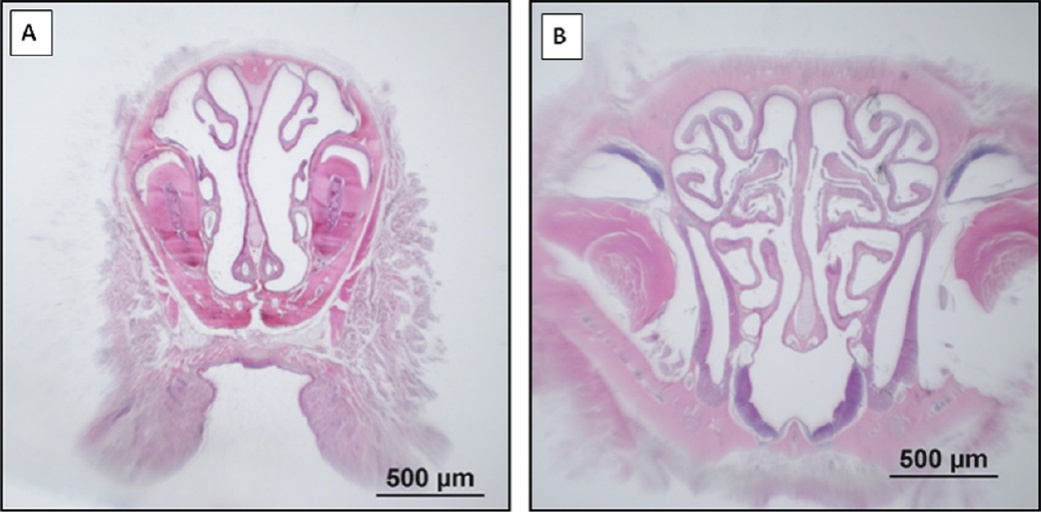
Localizations of nasal tissues selected for analysis. Representative light-microscopic images of H–E staining in selected nasal concha sections in the control group (A, B).

The histomorphological evaluation of nasal conchae of animals exposed to heated frying margarine for the control, acute, subacute, and chronic exposure groups was based on the H–E staining of sections (Figure 4).

#### 3.3.1 Control Group (*n* = 3)

The nasal conchae tissue of the control group showed a regular epithelial structure. Mucosal cavity structures were normal, and no increase was observed in inflammation and capillary permeability (Figure 4).

#### 3.3.2 Subacute Exposure Group (*n* = 7)

**Figure 5:**
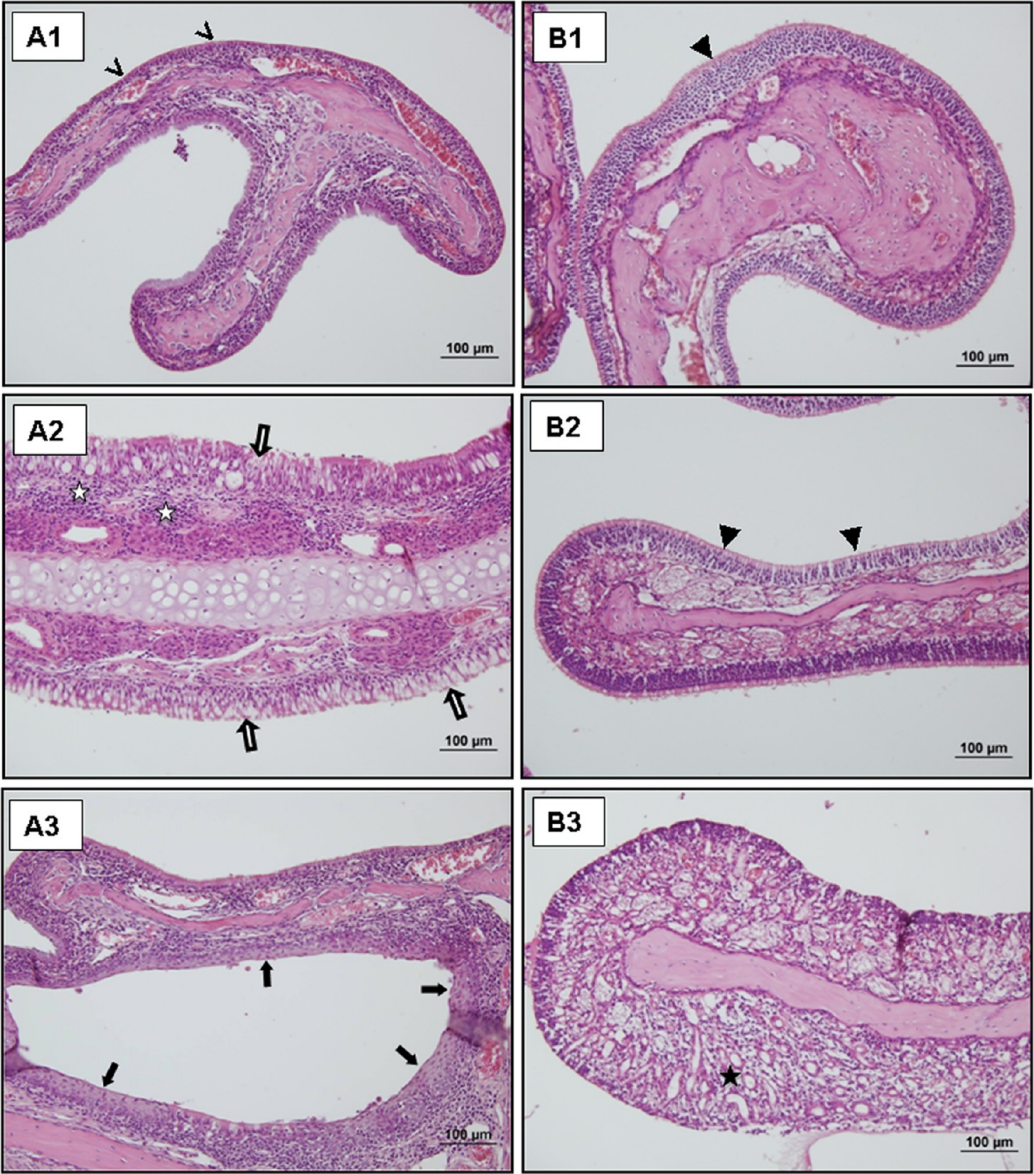
Representative light microscopic images of H–E staining in the nasal concha in the subacute group (**A1–3**) and the chronic group (**B1–3).** Inflammatory response of the nasal concha (
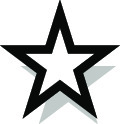
) and mucosal changes in the nasal concha, basal cell hyperplasia (▶), squamous metaplasia (
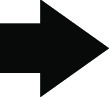
), goblet cell hyperplasia (
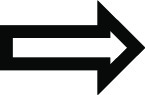
), and subepithelial region with granulation tissue (★).

Nasal mucosal changes, squamous metaplasia, and goblet cell hyperplasia were observed in this group, along with widespread mononuclear cell infiltration in the lamina propria. (Figure 5A1–3).

#### 3.3.3 Chronic Exposure Group (*n* = 7)

Higher damage was observed compared to the acute and subacute exposure groups. Basal cell hyperplasia in the epithelium, granulation in the subepithelial area, and diffuse mononuclear cell infiltration in the lamina propria were observed (Figure 5 B1–3).

## 4 Discussion

In this study, we found evidence supporting that acute, subacute, and long-term exposure to frying oil fumes in mice has detrimental effects on the tracheobronchial tree and lung parenchyma, starting from the nasal turbinates. In the nasal concha and tracheal mucosa, we detected degenerative changes in the epithelium in the acute exposure group, together with inflammation in the lamina propria and diffuse mononuclear cell infiltration in the tissue.

We found that mononuclear cell infiltration occurs rapidly at the lamina propria level with an increase in capillary number after acute mucosal injury, squamous metaplasia, and goblet cell hyperplasia with prolonged exposure, and granulation tissue development in the subepithelial area together with tissue repair mechanisms are activated. We also detected similar changes in the tracheal mucosa. Findings such as papillation and fibrosis supported the involvement of repair mechanisms in chronic exposure. Inflammation in the lung parenchyma was characterized by diffuse alveolar damage associated with diffuse hemorrhage, mononuclear cell infiltration, edema, and vascular congestion, as well as alveolar edema and thickening of the alveolar septa. These findings show that a single and acute exposure to the fumes of the frying oil used in our country has harmful effects on the respiratory parenchyma, as well as the nasal concha and trachea in mice. If the exposure continues, it leads to exacerbation of inflammation and activation of repair mechanisms leading to fibrosis in the tissue. In consequence, it is indicated that inflammation affecting the airways and parenchyma, which intensifies as the exposure time to frying smoke increases, may occur in workers of establishments that use the margarine for frying.

Previous studies have found evidence of severe obstructive airway disease associated with acute exposure to cooking oil fumes^41^ that lifetime, short-term, and low-level kitchen exposures in women aged over 65 years have been associated with respiratory complaints and pulmonary functional loss.^42^ The relationship between the respiratory effects^31,33−35^ associated with smoke exposure and frying oil exposure is consistent with our findings.

The importance of cellular changes and/or inflammatory markers associated with PAHs, aldehydes, and PM released during frying has been demonstrated,^16,17^ and findings supporting persistent oxidative stress in the airway epithelium were found in volunteers exposed to frying oil fumes.^43^ These findings were supported in an experimental study, and a clearer suggestion was presented regarding the mechanism of damage caused by exposure to frying oil fumes.^38^

It should be emphasized, however, that acute exposure to cooking oil fumes, as we have shown in our study, causes damage to all airways, starting from the nasal passages to the lung parenchyma. As the exposure time extends, the damage indicates that the repair mechanisms come into play.

A significant relationship was reported between the frying-time exposure of women older than 65 years and chronic respiratory symptoms, and the decrease in functional measures may be related to the respiratory effects of lifelong, short-time, and low-level kitchen exposures.^42^ Although limited, clinical evidence was reported on the respiratory effects of frying with different oils, types, and conditions based on workplace field studies.^31,33−35^ Simpson, Belfield, and Cooke^41^ reported a 22-year-old case of severe obstructive airway disease, which they called “obliterative bronchiolitis”, with no evidence of inflammation in the upper respiratory tract after acute exposure to vegetable oil fumes, following an epileptic seizure. This case shows that exposure to heavy frying oil fumes can lead to serious airway disorders; however, the pulmonary parenchymal effect was not fully revealed due to the lack of tissue analysis. The importance of cellular changes and/or inflammatory markers associated with PAHs, aldehydes, and PM emitted during frying has been shown.^16,17^ In addition, findings that support persistent oxidative stress in the airway epithelium were determined in volunteers exposed to frying oil fumes.^43^ The increase in the level of IL-1β after exposure to frying fumes was stressed as an indicator of the early inflammatory response.

Different from residential kitchens, workers are continuously exposed to frying fumes for regular and long durations in commercial kitchens where the strength of the emissions is also higher.^1^ The difference in emission strength was attributed to the differences in the style and amount of food.^7^ The detected compounds and their concentrations in a commercial kitchen’s indoor air in our previous study^37^ have the potential to cause the health effects reported in the literature.

PM_10_, a regulated criteria air pollutant, has been well-studied and known to have health effects. In terms of mass-based concentrations, PM_10_ consists mainly of PM_2.5_, which mainly consists of sub-micron particles (PM_1_), which mainly consist of UFP in terms of number concentration. Ma et al.^44,45^ investigated the effects of oil temperature (namely, starting and moderate smoke point temperatures) and time, both on mass and number concentrations, and proposed a two-way mitigation strategy: the use of lower oil volume and larger pans at relatively lower temperatures to primarily control particle number emissions, and the use of higher oil volumes and smaller pans at higher temperatures to mitigate particle mass emissions. Shi et al.^46^ showed that particle emissions from heated peanut oil had lognormal size distributions with spatially variable sizes decreasing with the distance from the source due to sharp cooling near the source and then volatilization of semivolatile organic compounds. Particles in frying fumes may be associated with adverse health effects. UFP have been found in biological media as single particles and/or as agglomerates. It has been suggested that when clustered UFP are given to the mice, the deficiency in cleaning mechanisms may result in inflammation, proliferation, fibrosis, and tumor formation in the lungs.^30^ UFP led to proinflammatory changes such as carbon and neutrophil accumulation, protein leakage, and glutathione modulation in studies that avoided high doses. In these studies, it has been demonstrated that the phagocytic activities of macrophages decrease and oxidative stress increases. Although the mechanisms of action for UFP have not been fully explained, the relationship between the extent of the surface area and inflammatory cellular activation has been emphasized.^27,30^

The exposure to PM_10_ concentrations was kept at the occupational levels by continuous monitoring, while the background levels were 40 to 2.5 times lower than the target concentration with an average of 4.6 times, in this study. However, a limitation of this study is the lack of UFP measurement. The detected inflammation in this study may have been related to any, measured or not, substance including UFP in the frying fumes. A relationship between particle concentrations and PAHs or aldehydes has not been reported in the literature. Therefore, it has been reported that they were not covariable factors responsible for the occurrence of adverse effects.^16^ In other words, substances such as aldehydes and PAHs or the complex interaction of all may also be responsible for the health risks associated with exposure to frying fumes.

## 5 Conclusions

The observation of significant inflammatory changes from acute to chronic exposures in the experimental model employed in this study indicates that the use of the vegetable margarine for frying poses occupational health risks to kitchen workers who are exposed to its fumes. Yet, the histopathological changes determined in our clinic with a diagnosis of alveolar damage in two occupational cases were similar to the findings of this study.

Acute and chronic inflammation, along with epithelial damage associated with fume exposure in the upper and lower airways, may underlie favorable conditions for the development of allergic respiratory diseases as well as peripheral airway diseases such as bronchiolitis obliterans and interstitial lung diseases.

The differences in characteristics of the frying margarine investigated in this study and those in the literature may be the determining factors of exposure content and magnitude, which requires continual investigation of frying oil characteristics, content, and toxicity of their fumes. In the meantime, monitoring the conditions of work, ensuring the presence of appropriate ventilation, and raising awareness among the workers in commercial kitchens are vital.

## Data Availability

The datasets generated and/or analyzed during the current study are available from the corresponding author upon reasonable request.

## Notes

The study protocol was approved by the Ethical Committee of Dokuz Eylül University Medical School (permit no: 31-2010).
